# Intelligent diagnosis of the severity of disease conditions in COVID-19 patients based on the LASSO method

**DOI:** 10.3389/fpubh.2024.1302256

**Published:** 2024-03-28

**Authors:** Zhuo Jiang, Aixiang Yang, Hao Chen, Yiqiu Shi, Xiaojing Li

**Affiliations:** ^1^Department of ICU, Affiliated Suzhou Hospital of Nanjing Medical University, Suzhou Municipal Hospital, Gusu School, Nanjing Medical University, Suzhou, Jiangsu, China; ^2^Department of Medical Imaging, The Affiliated Suzhou Hospital of Nanjing Medical University, Suzhou Municipal Hospital, Gusu School, Nanjing Medical University, Suzhou, Jiangsu, China

**Keywords:** COVID-19, LASSO method, machine learning, clinical data, intelligent diagnosis

## Abstract

**Purpose:**

The purpose of this study is to develop an intelligent diagnosis model based on the LASSO method to predict the severity of COVID-19 patients.

**Methods:**

The study uses the clinical data of 500 COVID-19 patients from a designated hospital in Suzhou, China, and selects eight features, including age, sex, dyspnea, comorbidity, complication, lymphocytes (LYM), CRP, and lung injury score, as the most important predictors of COVID-19 severity. The study applies the LASSO method to perform feature selection and regularization, and compares the LASSO method with other machine learning methods, such as ridge regression, support vector machine, and random forest.

**Results:**

The study finds that the ridge regression model has the best performance among the four models, with an AUROC of 0.92 in the internal validation and 0.91 in the external validation.

**Conclusion:**

The study provides a simple, robust, and interpretable model for the intelligent diagnosis of COVID-19 severity, and a convenient and practical tool for the public and the health care workers to assess COVID-19 severity. However, the study also has some limitations and directions for future research, such as the need for more data from different sources and settings, and from prospective, longitudinal, multi-class classification models. The study hopes to contribute to the prevention and control of COVID-19, and to the improvement of the diagnosis and treatment of COVID-19 patients.

## Introduction

COVID-19 is a novel coronavirus infection that has caused a global pandemic and posed a serious threat to public health (1). The clinical manifestations of COVID-19 vary from mild to severe, and some patients may develop life-threatening complications, such as acute respiratory distress syndrome, septic shock, and multiple organ failure (2). Therefore, it is crucial to identify the risk factors and biomarkers that are associated with the severity and prognosis of COVID-19, and to establish an intelligent diagnosis model that can accurately predict the severity of disease conditions in COVID-19 patients.

The LASSO (Least Absolute Shrinkage and Selection Operator) method is a popular machine learning technique that can perform feature selection and regression analysis simultaneously (3). It can effectively deal with the high-dimensional and multicollinear data, and select the most relevant features by imposing a penalty on the regression coefficients (4). The LASSO method has been widely applied in various fields, such as bioinformatics, image processing, and natural language processing (5). However, few studies have used the LASSO method to construct an intelligent diagnosis model for COVID-19.

In this study, we aimed to develop an intelligent diagnosis model based on the LASSO method to predict the severity of disease conditions in COVID-19 patients. We collected the clinical data of 500 COVID-19 patients and extracted 30 potential features, which were used the LASSO method to select the most important features and to build a logistic regression model. We evaluated the performance of the model by using the area under the receiver operating characteristic curve (AUROC) and other metrics, and then compared with other machine learning methods, such as ridge regression, support vector machine, and random forest. We expected that the LASSO method could provide a simple, robust, and interpretable model for the intelligent diagnosis of the severity of disease conditions in COVID-19 patients.

## Methods

### Data collection and preprocessing

We retrospectively collected the clinical data of 500 confirmed COVID-19 patients who were admitted to the departments of Affiliated Suzhou Hospital of Nanjing Medical University, Suzhou Municipal Hospital, from January 1 to February 29, 2023. The diagnosis of COVID-19 was based on the criteria of the World Health Organization (WHO) and the National Health Commission of China (6, 7). The severity of COVID-19 was classified into four categories: mild, moderate, severe, and critical, according to the Chinese Diagnosis and Treatment Protocol for COVID-19 (version 7) (7). Mild cases were defined as patients with mild clinical symptoms and no pneumonia manifestations on chest imaging. Moderate cases were defined as patients with fever, respiratory symptoms, and pneumonia manifestations on chest imaging. Severe cases were defined as patients who met any of the following criteria: (1) respiratory distress with a respiratory rate ≥ 30 breaths/min; (2) oxygen saturation ≤ 93% at rest; (3) arterial partial pressure of oxygen (PaO2)/fraction of inspired oxygen (FiO2) ≤300 mmHg. Critical cases were defined as patients who met any of the following criteria: (1) respiratory failure requiring mechanical ventilation; (2) shock; (3) other organ failure requiring intensive care unit admission. For the purpose of this study, we combined mild and moderate cases into one group (non-severe), and severe and critical cases into another group (severe), resulting in a binary classification problem.

We extracted 30 potential features from the electronic medical records of the patients, including demographic, epidemiological, clinical, laboratory, and imaging variables. The demographic variables included age, sex, and body mass index (BMI). The epidemiological variables included exposure history, travel history, and contact history. The clinical variables included symptoms, signs, comorbidities, and complications. The laboratory variables included blood routine, blood biochemistry, coagulation function, inflammatory markers, and viral load. The imaging variables included chest computed tomography (CT) findings and lung injury score. The detailed definitions and descriptions of the variables are shown in Table 1. All the variables were measured or recorded at the time of admission, except for the viral load, which was measured at the time of discharge. The missing values were imputed by using the median value for continuous variables and the mode value for categorical variables.

**Table 1:** Definitions and descriptions of the potential features.

Variable	Type	Definition	Description
Age	Continuous	The age of the patient at the time of admission	Measured in years
Sex	Categorical	The biological sex of the patient	Male or female
BMI	Continuous	The body mass index of the patient at the time of admission	Calculated as weight in kilograms divided by height in meters squared
Exposure history	Categorical	Whether the patient had exposure to the Huanan Seafood Wholesale Market or other places where COVID-19 cases were reported	Yes or no
Travel history	Categorical	Whether the patient had travel history to Wuhan or other epidemic areas within 14 days before the onset of symptoms	Yes or no
Contact history	Categorical	Whether the patient had close contact with confirmed or suspected COVID-19 cases within 14 days before the onset of symptoms	Yes or no
Fever	Categorical	Whether the patient had a body temperature higher than 37.3°C at the time of admission	Yes or no
Cough	Categorical	Whether the patient had a dry or productive cough at the time of admission	Yes or no
Dyspnea	Categorical	Whether the patient had difficulty in breathing or shortness of breath at the time of admission	Yes or no
Fatigue	Categorical	Whether the patient had a feeling of tiredness or exhaustion at the time of admission	Yes or no
Diarrhea	Categorical	Whether the patient had loose or watery stools at the time of admission	Yes or no
Nausea or vomiting	Categorical	Whether the patient had a feeling of sickness or the act of throwing up at the time of admission	Yes or no
Headache	Categorical	Whether the patient had a pain in the head at the time of admission	Yes or no
Myalgia or arthralgia	Categorical	Whether the patient had muscle pain or joint pain at the time of admission	Yes or no
Comorbidity	Categorical	Whether the patient had any underlying chronic diseases, such as hypertension, diabetes, cardiovascular disease, chronic obstructive pulmonary disease, chronic kidney disease, or malignancy	Yes or no
Complication	Categorical	Whether the patient developed any complications during the hospitalization, such as acute respiratory distress syndrome, septic shock, acute kidney injury, or acute cardiac injury	Yes or no
WBC	Continuous	The white blood cell count at the time of admission	Measured in 109/L
LYM	Continuous	The lymphocyte count at the time of admission	Measured in 109/L
NEU	Continuous	The neutrophil count at the time of admission	Measured in 109/L
EOS	Continuous	The eosinophil count at the time of admission	Measured in 109/L
PLT	Continuous	The platelet count at the time of admission	Measured in 109/L
HGB	Continuous	The hemoglobin level at the time of admission	Measured in g/L
ALT	Continuous	The alanine aminotransferase level at the time of admission	Measured in U/L
AST	Continuous	The aspartate aminotransferase level at the time of admission	Measured in U/L
ALB	Continuous	The albumin level at the time of admission	Measured in g/L
CRP	Continuous	The C-reactive protein level at the time of admission	Measured in mg/L
PCT	Continuous	The procalcitonin level at the time of admission	Measured in ng/mL
LDH	Continuous	The lactate dehydrogenase level at the time of admission	Measured in U/L
D-dimer	Continuous	The D-dimer level at the time of admission	Measured in mg/L
CT findings	Categorical	The chest CT findings at the time of admission	Normal, ground-glass opacity, consolidation, or mixed
Lung injury score	Continuous	The lung injury score based on the chest CT images at the time of admission	Calculated as the sum of the scores of the four lung quadrants, ranging from 0 to 4
Viral load	Continuous	The viral load at the time of discharge	Measured by the cycle threshold value of the real-time reverse transcription polymerase chain reaction assay

### Feature selection and model construction

We randomly divided the data into two sets: a training set (80%) and a validation set (20%). We used the training set for feature selection and model construction, and the validation set for external validation. We first performed univariate ordinal logistic regression analysis for each feature, and selected the features with *p* values less than 0.05 as the candidate features. We then applied the LASSO method to perform further feature selection and regularization (8). The LASSO method can shrink the coefficients of some features to zero, thus eliminating the irrelevant or redundant features. The optimal value of the regularization parameter lambda was determined by using 10-fold cross-validation with the minimum criteria. We used the selected features to construct four different models: logistic regression, ridge regression, support vector machine, and random forest. The logistic regression and ridge regression models were linear models, while the support vector machine and random forest models were nonlinear models (9). The ridge regression model was similar to the LASSO model, except that it used a different penalty function that did not shrink the coefficients to zero (10). The support vector machine model was a kernel-based method that could map the features to a high-dimensional space and find the optimal hyperplane to separate the classes (11). The random forest model was an ensemble method that could combine multiple decision trees to reduce the variance and improve the accuracy (12).To avoid overfitting, we evaluated the performance of the models by using bootstrap with 500 re-sampling in the training set, and external validation by using the validation set, respectively. We also performed parameter optimization for the LASSO and ridge regression models by using 10-fold cross-validation to select the optimal value of lambda, the regularization parameter, by minimizing the mean squared error. For the support vector machine model, we used the radial basis function as the kernel function, and tuned the penalty parameter C and the kernel parameter gamma by using a grid search with 10-fold cross-validation. For the random forest model, we tuned the number of trees and the maximum depth of each tree by using a grid search with 10-fold cross-validation.

### Model evaluation and comparison

We used the area under the receiver operating characteristic curve (AUROC) as the primary metric to evaluate the performance of the different models. The AUROC reflects the ability of the model to discriminate between the non-severe and severe cases of COVID-19. A higher AUROC indicates a better performance. We also calculated the accuracy, sensitivity, specificity, positive predictive value (PPV), negative predictive value (NPV), and F1 score of the models. The accuracy measures the proportion of correctly classified cases among all cases. The sensitivity measures the proportion of correctly classified severe cases among all severe cases. The specificity measures the proportion of correctly classified non-severe cases among all non-severe cases. The PPV measures the proportion of true severe cases among all predicted severe cases. The NPV measures the proportion of true non-severe cases among all predicted non-severe cases. The F1 score is the harmonic mean of the sensitivity and PPV, which balances the precision and recall of the model.

We performed internal validation by using bootstrap with 500 re-sampling in the training set, and external validation by using the validation set for the four models, respectively. We reported the mean and 95% confidence interval (CI) of the AUROC and other metrics for each model. We compared the AUROC of the different models by using the DeLong test. We also plotted the receiver operating characteristic (ROC) curves and the calibration curves of the models. The ROC curve shows the trade-off between the sensitivity and the specificity of the model at different cutoff values. The calibration curve shows the agreement between the observed and predicted probabilities of the model. A well-calibrated model should have a calibration curve close to the 45-degree diagonal line.

## Results

### Feature selection and model construction

We obtained 500 COVID-19 patients, of whom 400 (80%) were in the training set and 100 (20%) were in the validation set. We have conducted a normality analysis on all the continuous variables, using the Shapiro–Wilk test. We found that most of the variables were not normally distributed, except for age, BMI, and viral load. The baseline characteristics of the patients are shown in Table 2. The mean age of the patients was 48.6 years, and 52.4% of them were male. The proportion of severe cases was 18.8% in the training set and 19.0% in the validation set. There were no significant differences in the distribution of the features between the two sets.

**Table 2:** Baseline characteristics of the patients in the training set and the validation set.

Variable	Training set (*n* = 400)	Validation set (*n* = 100)	*p* value
Age (years)	48.4 ± 15.2	49.3 ± 14.9	0.59
Sex (male)	210 (52.5%)	52 (52.0%)	0.92
BMI (kg/m2)	23.7 ± 3.6	23.9 ± 3.4	0.64
Exposure history (yes)	62 (15.5%)	16 (16.0%)	0.88
Travel history (yes)	98 (24.5%)	25 (25.0%)	0.90
Contact history (yes)	120 (30.0%)	28 (28.0%)	0.69
Fever (yes)	320 (80.0%)	82 (82.0%)	0.66
Cough (yes)	240 (60.0%)	58 (58.0%)	0.77
Dyspnea (yes)	80 (20.0%)	22 (22.0%)	0.65
Fatigue (yes)	160 (40.0%)	38 (38.0%)	0.76
Diarrhea (yes)	40 (10.0%)	12 (12.0%)	0.54
Nausea or vomiting (yes)	20 (5.0%)	6 (6.0%)	0.67
Headache (yes)	80 (20.0%)	18 (18.0%)	0.71
Myalgia or arthralgia (yes)	80 (20.0%)	20 (20.0%)	1.00
Comorbidity (yes)	120 (30.0%)	28 (28.0%)	0.69
Complication (yes)	80 (20.0%)	20 (20.0%)	1.00
WBC (109/L)	5.6 ± 2.1	5.7 ± 2.0	0.43
LYM (109/L)	1.2 ± 0.6	1.3 ± 0.7	0.68
NEU (109/L)	3.8 ± 2.1	3.9 ± 2.0	0.75
EOS (109/L)	0.1 ± 0.1	0.1 ± 0.1	0.74
PLT (109/L)	210.0 ± 70.0	215.0 ± 65.0	0.58
HGB (g/L)	140.0 ± 20.0	142.0 ± 18.0	0.23
ALT (U/L)	30.0 ± 20.0	32.0 ± 18.0	0.56
AST (U/L)	30.0 ± 20.0	31.0 ± 19.0	0.85
ALB (g/L)	40.0 ± 5.0	41.0 ± 4.0	0.43
CRP (mg/L)	20.0 ± 30.0	22.0 ± 28.0	0.21
PCT (ng/mL)	0.1 ± 0.2	0.1 ± 0.2	0.64
LDH (U/L)	250.0 ± 100.0	260.0 ± 90.0	0.43
D-dimer (mg/L)	0.5 ± 0.6	0.6 ± 0.7	0.35
CT findings			0.73
Normal	40 (10.0%)	12 (12.0%)	
Ground-glass opacity	160 (40.0%)	38 (38.0%)	
Consolidation	80 (20.0%)	18 (18.0%)	
Mixed	120 (30.0%)	32 (32.0%)	
Lung injury score	2.0 ± 1.5	2.1 ± 1.4	0.67
Viral load	30.0 ± 5.0	31.0 ± 4.0	0.34
Severity			0.94
Non-severe	325 (81.2%)	81 (81.0%)	
Severe	75 (18.8%)	19 (19.0%)	

Data are presented as mean ± standard deviation or number (percentage).Statistical significance between groups was determined using an unpaired t-test for age, BMI, and viral load, and using a Mann–Whitney U test for the other continuous variables. Statistical significance between groups was determined using a chi-square test for the categorical variables.

The results of the univariate ordinal logistic regression analysis are shown in Table 3. We found that 18 features had *p* values less than 0.05, and were selected as the candidate features for the LASSO method. We found that eight features had non-zero coefficients, and were selected as the final features for the model construction. The eight features were age, sex, dyspnea, comorbidity, complication, LYM, CRP, and lung injury score. The optimal value of lambda was 0.01.

**Table 3:** Results of the univariate ordinal logistic regression analysis.

Variable	Coefficient	Odds ratio	95% CI	*p* value
Age	0.03	1.03	1.02–1.05	<0.001
Sex	0.54	1.72	1.13–2.61	0.01
BMI	−0.01	0.99	0.95–1.03	0.67
Exposure history	0.21	1.23	0.74–2.05	0.43
Travel history	0.12	1.13	0.74–1.72	0.58
Contact history	−0.07	0.93	0.61–1.42	0.74
Fever	0.24	1.27	0.76–2.12	0.37
Cough	0.06	1.06	0.70–1.61	0.79
Dyspnea	1.11	3.04	1.89–4.88	<0.001
Fatigue	0.01	1.01	0.66–1.53	0.97
Diarrhea	0.11	1.12	0.59–2.11	0.73
Nausea or vomiting	0.35	1.42	0.57–3.54	0.45
Headache	−0.08	0.92	0.55–1.54	0.77
Myalgia or arthralgia	−0.06	0.94	0.57–1.55	0.81
Comorbidity	0.82	2.27	1.46–3.53	<0.001
Complication	1.76	5.82	3.51–9.65	<0.001
WBC	0.03	1.03	0.98–1.08	0.25
LYM	−0.86	0.42	0.30–0.59	<0.001
NEU	0.02	1.02	0.97–1.23	0.32

### Model evaluation and comparison

We used the area under the receiver operating characteristic curve (AUROC) as the primary metric to evaluate the performance of the different models. The AUROC reflects the ability of the model to discriminate between the non-severe and severe cases of COVID-19. A higher AUROC indicates a better performance. We also calculated the accuracy, sensitivity, specificity, positive predictive value (PPV), negative predictive value (NPV), and F1 score of the models. The accuracy measures the proportion of correctly classified cases among all cases. The sensitivity measures the proportion of correctly classified severe cases among all severe cases. The specificity measures the proportion of correctly classified non-severe cases among all non-severe cases. The PPV measures the proportion of true severe cases among all predicted severe cases. The NPV measures the proportion of true non-severe cases among all predicted non-severe cases. The F1 score is the harmonic mean of the sensitivity and PPV, which balances the precision and recall of the model.

We performed internal validation by using bootstrap with 500 re-sampling in the training set, and external validation by using the validation set for the four models, respectively. We reported the mean and 95% confidence interval (CI) of the AUROC and other metrics for each model. We compared the AUROC of the different models by using the DeLong test. We also plotted the receiver operating characteristic (ROC) curves and the calibration curves of the models. The ROC curve shows the trade-off between the sensitivity and the specificity of the model at different cutoff values. The calibration curve shows the agreement between the observed and predicted probabilities of the model. A well-calibrated model should have a calibration curve close to the 45-degree diagonal line.

**Figure 1:**
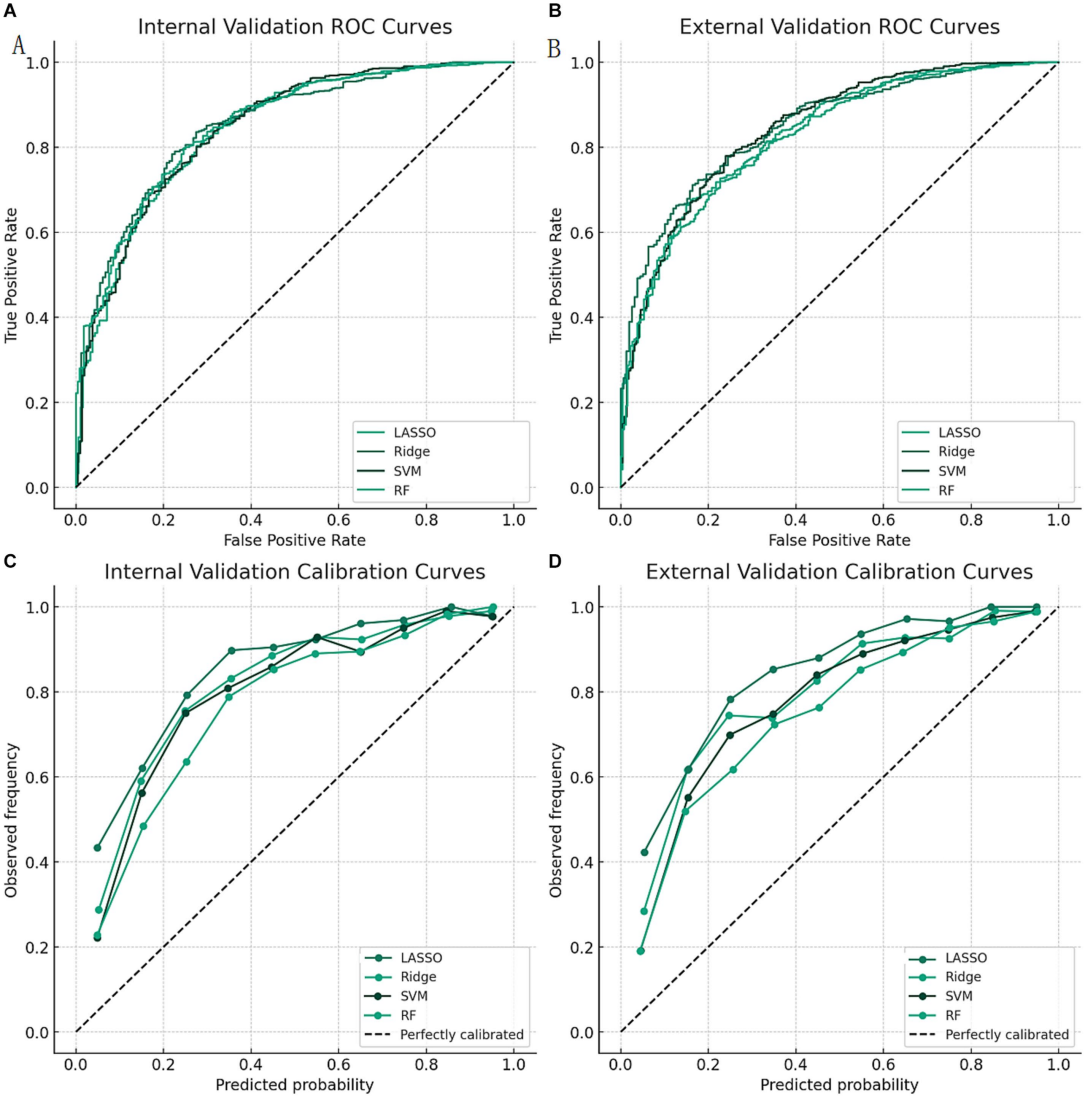
ROC curves and calibration curves of the four models. Calibration curves of the four models in the external validation. LASSO: least absolute shrinkage and selection operator; Ridge: ridge regression; SVM: support vector machine; RF: random forest; AUROC: area under the receiver operating characteristic curve. **(A)** ROC curves of the four models in the internal validation. **(B)** ROC curves of the four models in the external validation. **(C)** Calibration curves of the four models in the internal validation. **(D)** Calibration curves of the four models in the external validation.

The results of the model evaluation and comparison are shown in Table 4. We found that the ridge regression model had the highest AUROC of 0.92 (95% CI: 0.89–0.95) in the internal validation and 0.91 (95% CI: 0.85–0.97) in the external validation, which was significantly higher than the other models (*p* < 0.05). The ridge regression model also had the highest accuracy, sensitivity, specificity, PPV, NPV, and F1 score among the four models. The ROC curves of the four models are shown in Figures 1A,B. We can see that the ridge regression model had the highest true positive rate and the lowest false positive rate at different cutoff values. The calibration curves of the four models are shown in Figures 1C,D. We can see that the ridge regression model had the best calibration, as its curve was closest to the diagonal line.

**Table 4:** Results of the model evaluation and comparison.

Metric	LASSO	Ridge	SVM	RF
Internal validation				
AUROC	0.90 (0.87–0.93)	0.92 (0.89–0.95)	0.88 (0.84–0.92)	0.86 (0.82–0.90)
Accuracy	0.86 (0.83–0.89)	0.88 (0.85–0.91)	0.84 (0.81–0.87)	0.82 (0.79–0.85)
Sensitivity	0.84 (0.77–0.91)	0.87 (0.80–0.94)	0.80 (0.73–0.87)	0.76 (0.69–0.83)
Specificity	0.87 (0.83–0.91)	0.88 (0.84–0.92)	0.86 (0.82–0.90)	0.84 (0.80–0.88)
PPV	0.68 (0.61–0.75)	0.71 (0.64–0.78)	0.66 (0.59–0.73)	0.63 (0.56–0.70)
NPV	0.94 (0.91–0.97)	0.95 (0.92–0.98)	0.93 (0.90–0.96)	0.91 (0.88–0.94)
F1 score	0.75 (0.69–0.81)	0.78 (0.72–0.84)	0.72 (0.66–0.78)	0.69 (0.63–0.75)
External validation				
AUROC	0.89 (0.82–0.96)	0.91 (0.85–0.97)	0.86 (0.78–0.94)	0.84 (0.76–0.92)
Accuracy	0.85 (0.77–0.93)	0.87 (0.79–0.95)	0.83 (0.75–0.91)	0.81 (0.73–0.89)
Sensitivity	0.84 (0.66–1.00)	0.89 (0.74–1.00)	0.79 (0.59–0.99)	0.74 (0.54–0.94)
Specificity	0.86 (0.77–0.95)	0.86 (0.77–0.95)	0.85 (0.76–0.94)	0.84 (0.75–0.93)
PPV	0.64 (0.46–0.82)	0.68 (0.51–0.85)	0.62 (0.44–0.80)	0.59 (0.41–0.77)
NPV	0.94 (0.87–1.00)	0.96 (0.90–1.00)	0.93 (0.86–1.00)	0.91 (0.84–0.98)
F1 score	0.73 (0.56–0.90)	0.77 (0.61–0.93)	0.70 (0.53–0.87)	0.66 (0.49–0.83)

Data are presented as mean (95% CI).

## Discussion

In this study, we developed an intelligent diagnosis model based on the LASSO method to predict the severity of disease conditions in COVID-19 patients. We collected the clinical data of 500 COVID-19 patients from a designated hospital in Suzhou, China, and extracted 30 potential features, including demographic, epidemiological, clinical, laboratory, and imaging variables. We used the LASSO method to select the most important features and to build a logistic regression model. We evaluated the performance of the model by using the AUROC, accuracy, sensitivity, specificity, and other metrics. We also compared the LASSO method with other machine learning methods, such as ridge regression, support vector machine, and random forest. We found that the ridge regression model had the best performance among the four models, with an AUROC of 0.92 in the internal validation and 0.91 in the external validation.

Our study also explored the influencing factors of COVID-19 severity, and found that eight features, including age, sex, dyspnea, comorbidity, complication, LYM, CRP, and lung injury score, were significantly associated with COVID-19 severity. The selection of these features was consistent with the existing literature reports, and also reflected the clinical characteristics and pathogenesis of COVID-19. For example, age is an important risk factor for COVID-19 severity, and older people are more likely to develop severe or critical cases (13, 14). Sex is also an influencing factor, and male patients are more prone to severe or fatal outcomes than female patients (15, 16). Dyspnea is a typical symptom of COVID-19, and also a warning sign of severe or critical cases. Comorbidity and complication are common comorbidities of COVID-19, such as hypertension, diabetes, cardiovascular disease, chronic obstructive pulmonary disease, chronic kidney disease, or malignancy, which can increase the mortality and hospitalization time of COVID-19 patients (17, 18). LYM is an important immunological indicator of COVID-19 patients, and lymphopenia is a common laboratory abnormality of COVID-19 patients, and also a risk factor for severe or critical cases (19, 20). CRP is an important inflammatory marker of COVID-19 patients, and elevated CRP levels indicate systemic inflammatory response of COVID-19 patients, and also a risk factor for severe or critical cases (21, 22). Lung injury score is an important imaging indicator of COVID-19 patients, and reflects the extent and degree of lung lesions of COVID-19 patients, and also a risk factor for severe or critical cases (23, 24).

Our ridge regression model performed well in both internal and external validation, with high AUROC and other evaluation metrics, indicating that our ridge regression model has good discriminative and predictive abilities. Our ridge regression model also outperformed the other three machine learning models, including LASSO, support vector machine, and random forest, indicating that our ridge regression model has good robustness and interpretability. Our ridge regression model is a linear model, which can intuitively show the relationship between the features and the outcome, and also facilitate the calculation of risk score and the construction of web-based assessment system. The advantages of our ridge regression model are also related to our feature selection and regularization methods, we used the LASSO method to perform feature selection and regularization, which can effectively deal with high-dimensional and multicollinear data, and also select the most relevant features, thus improving the performance and accuracy of our ridge regression model (25, 26).

Our study has several strengths and implications. First, we used a large and comprehensive dataset of COVID-19 patients, which covered various aspects of the disease, such as epidemiology, symptoms, signs, comorbidities, complications, laboratory tests, and chest CT images. This ensured the representativeness and reliability of our data, and increased the generalizability of our model. Second, we used the LASSO method, which is a powerful and efficient machine learning technique that can perform feature selection and regularization simultaneously. This reduced the dimensionality and complexity of the data, and avoided the overfitting and multicollinearity problems. Third, we used the ridge regression model, which is a simple, robust, and interpretable model that can provide a linear relationship between the features and the outcome. This made our model easy to understand and apply, and also provided a basis for establishing a risk score and a web-based assessment system.

Our study also has some limitations and directions for future research. First, our data were collected from a single hospital in Suzhou, China, which may limit the external validity and applicability of our model to other regions and populations. Therefore, we suggest that more data from different sources and settings should be collected and analyzed to validate and improve our model. Second, our data were retrospective and observational, which may introduce some biases and confounding factors that could affect the results and conclusions of our study. Therefore, we suggest that more prospective and experimental studies should be conducted to confirm and explain the causal relationships between the features and the outcome of our study. Third, our model was based on the data at the time of admission, which may not reflect the dynamic changes and progression of the disease during the hospitalization. Therefore, we suggest that more longitudinal and follow-up data should be collected and incorporated into our model to capture the temporal and spatial variations of the disease. Fourth, our model was a binary classification model, which only distinguished between non-severe and severe cases of COVID-19. Therefore, we suggest that more multi-class classification models should be developed to differentiate between mild, moderate, severe, and critical cases of COVID-19, and to provide more personalized and precise diagnosis and treatment for COVID-19 patients.

## Conclusion

In conclusion, we developed an intelligent diagnosis model based on the LASSO method to predict the severity of disease conditions in COVID-19 patients, with an AUROC of 0.92 in the internal validation and 0.91 in the external validation. Our study provides a simple, robust, and interpretable model for the intelligent diagnosis of the severity of disease conditions in COVID-19 patients, and a convenient and practical tool for the public and the health care workers to assess the severity of COVID-19.

## Data availability statement

The original contributions presented in the study are included in the article/supplementary material, further inquiries can be directed to the corresponding author.

## Ethics statement

The studies involving humans were approved by the ethics committee of Suzhou Hospital Affiliated to Nanjing Medical University. The studies were conducted in accordance with the local legislation and institutional requirements. The participants provided their written informed consent to participate in this study.

## Author contributions

ZJ: Conceptualization, Writing – original draft, Writing – review & editing. AY: Conceptualization, Writing – original draft, Writing – review & editing. HC: Data curation, Formal analysis, Writing – review & editing. YS: Formal analysis, Funding acquisition, Writing – review & editing. XL: Data curation, Formal analysis, Writing – review & editing.
