# Corrigendum: Intelligent diagnosis of the severity of disease conditions in COVID-19 patients based on the LASSO method

**DOI:** 10.3389/fpubh.2024.1421217

**Published:** 2024-05-06

**Authors:** Zhuo Jiang, Aixiang Yang, Hao Chen, Yiqiu Shi, Xiaojing Li

**Affiliations:** ^1^Department of ICU, Affiliated Suzhou Hospital of Nanjing Medical University, Suzhou Municipal Hospital, Gusu School, Nanjing Medical University, Suzhou, Jiangsu, China; ^2^Department of Medical Imaging, The Affiliated Suzhou Hospital of Nanjing Medical University, Suzhou Municipal Hospital, Gusu School, Nanjing Medical University, Suzhou, Jiangsu, China

**Keywords:** COVID-19, LASSO method, machine learning, clinical data, intelligent diagnosis

In the published article, there was an error in the **Abstract** section, Methods. This sentence previously stated:

“Methods: The study uses the clinical data of 500 COVID-19 patients from a designated hospital in Guangzhou, China, and selects eight features, including age, sex, dyspnea, comorbidity, complication, lymphocytes (LYM), CRP, and lung injury score, as the most important predictors of COVID-19 severity.”

The corrected sentence appears below:

“Methods: The study uses the clinical data of 500 COVID-19 patients from a designated hospital in Suzhou, China, and selects eight features, including age, sex, dyspnea, comorbidity, complication, lymphocytes (LYM), CRP, and lung injury score, as the most important predictors of COVID-19 severity.”

In the published article, there was also an error in the **Discussion** section, paragraph 1. This sentence previously stated:

“In this study, we developed an intelligent diagnosis model based on the LASSO method to predict the severity of disease conditions in COVID-19 patients. We collected the clinical data of 500 COVID-19 patients from a designated hospital in Guangzhou, China, and extracted 30 potential features, including demographic, epidemiological, clinical, laboratory, and imaging variables.”

The corrected sentence appears below:

“In this study, we developed an intelligent diagnosis model based on the LASSO method to predict the severity of disease conditions in COVID-19 patients. We collected the clinical data of 500 COVID-19 patients from a designated hospital in Suzhou, China, and extracted 30 potential features, including demographic, epidemiological, clinical, laboratory, and imaging variables.”

A correction has been made to the **Discussion section**, paragraph 5. This sentence previously stated:

“Our study also has some limitations and directions for future research. First, our data were collected from a single hospital in Guangzhou, China, which may limit the external validity and applicability of our model to other regions and populations.”

The corrected sentence appears below:

“Our study also has some limitations and directions for future research. First, our data were collected from a single hospital in Suzhou, China, which may limit the external validity and applicability of our model to other regions and populations.”

The authors apologize for this error and state that this does not change the scientific conclusions of the article in any way. The original article has been updated.

